# Airway-Predominant Mucous Membrane Pemphigoid Causing Recurrent Central Airway Obstruction: A Fatal Case and Review of Interventional Management

**DOI:** 10.7759/cureus.99001

**Published:** 2025-12-11

**Authors:** Victor A Perez-Gutierrez, Lein Dalati, Abduljaba Adi, Jefferson Chambers, Sikandar Ansari

**Affiliations:** 1 Pulmonary and Critical Care Medicine, Huntsman Cancer Institute, University of Utah, Salt Lake City, USA; 2 Medicine, Bolu Abant Izzet Baysal Medical School, Bolu, TUR; 3 Medicine, Bașkent University, Ankara, TUR

**Keywords:** airway stenosis, autoimmune blistering disease, central airway obstruction, interventional bronchoscopy, mucous membrane pemphigoid

## Abstract

Mucous membrane pemphigoid (MMP) is a chronic autoimmune blistering disorder that primarily affects mucosal surfaces. Although laryngeal and tracheal involvement occurs less frequently, airway disease can lead to irreversible cicatricial stenosis and life-threatening obstruction. Lower airway involvement is rare but increasingly recognized in the emerging entity termed "pemphigoid of the pulmonary system" (POPS). We report a 62-year-old man with remote, biopsy-proven MMP who developed progressive tracheobronchial involvement after years of clinical remission. He presented with worsening dyspnea, hemoptysis, and recurrent mucositis. Imaging demonstrated complete opacification of one hemithorax with mediastinal shift secondary to central airway obstruction. Bronchoscopy revealed severe subglottic stenosis, friable tracheobronchial mucosa, and near-complete occlusion of the left main bronchus by circumferential fibrotic membranes. Balloon dilation, cryotherapy, and mechanical debridement produced transient airway patency, but rapid restenosis recurred. Direct immunofluorescence from the oral mucosa confirmed an immunoglobulin A (IgA-predominant MMP. Despite high-dose corticosteroids and repeated bronchoscopic interventions, the patient developed refractory hypoxemic respiratory failure and died. Airway-predominant MMP is an uncommon but aggressive phenotype characterized by mucosal inflammation, friability, and rapidly progressive scarring. Diagnostic delays are common because airway biopsies often lack an intact basement membrane, and serologies are frequently negative or low-titer. Interventional bronchoscopy is central to management; balloon dilation and cryotherapy offer temporary relief, but restenosis is expected due to the underlying autoimmune fibrosis. Mortality remains high when airway involvement is recognized late, as reflected in the POPS case series. This case demonstrates the destructive potential of airway-predominant MMP and the importance of early recognition, prompt bronchoscopy, and multidisciplinary management. Clinicians should maintain a high index of suspicion for airway involvement in patients with recurrent mucosal disease or unexplained respiratory symptoms to prevent irreversible stenosis.

## Introduction

Mucous membrane pemphigoid (MMP) is a chronic autoimmune subepithelial blistering disorder that primarily affects mucosal surfaces, including the oral cavity, conjunctiva, larynx and upper airway, esophagus, and genital mucosa, with less frequent skin involvement [1]. Autoantibodies directed against basement membrane zone (BMZ) components, including BP180 (type XVII collagen), BP230, laminin-332, and the integrin α6β4 complex, activate complement and trigger inflammatory cascades that lead to subepithelial blistering and progressive scarring [2-4]. In contrast to bullous pemphigoid, MMP often involves multiple mucosal sites, may display both IgG and IgA deposition, and carries a higher risk of irreversible fibrosis contributing to substantial morbidity such as ocular scarring or airway compromise [1]. Laryngeal and tracheal involvement occurs in fewer than 10% of MMP cases but may lead to severe airway stenosis and life-threatening obstruction. Lower airway disease is even rarer but increasingly recognized. A recent review of “pemphigoid of the pulmonary system” (POPS) identified tracheal, bronchial, and occasional parenchymal involvement associated with high morbidity and mortality [5,6]. Expression of BMZ antigens such as BP180 and laminin-332 within the respiratory epithelium provides a mechanistic basis for direct autoimmune injury to the lower airway. Nevertheless, airway stenosis remains the most dramatic and urgent pulmonary complication of MMP [6]. We report a case of long-standing MMP complicated by rapidly progressive central airway obstruction, recurrent bronchoscopic interventions, and ultimately fatal respiratory failure, underscoring the critical importance of early recognition and multidisciplinary management.

## Case presentation

**Figure 1:**
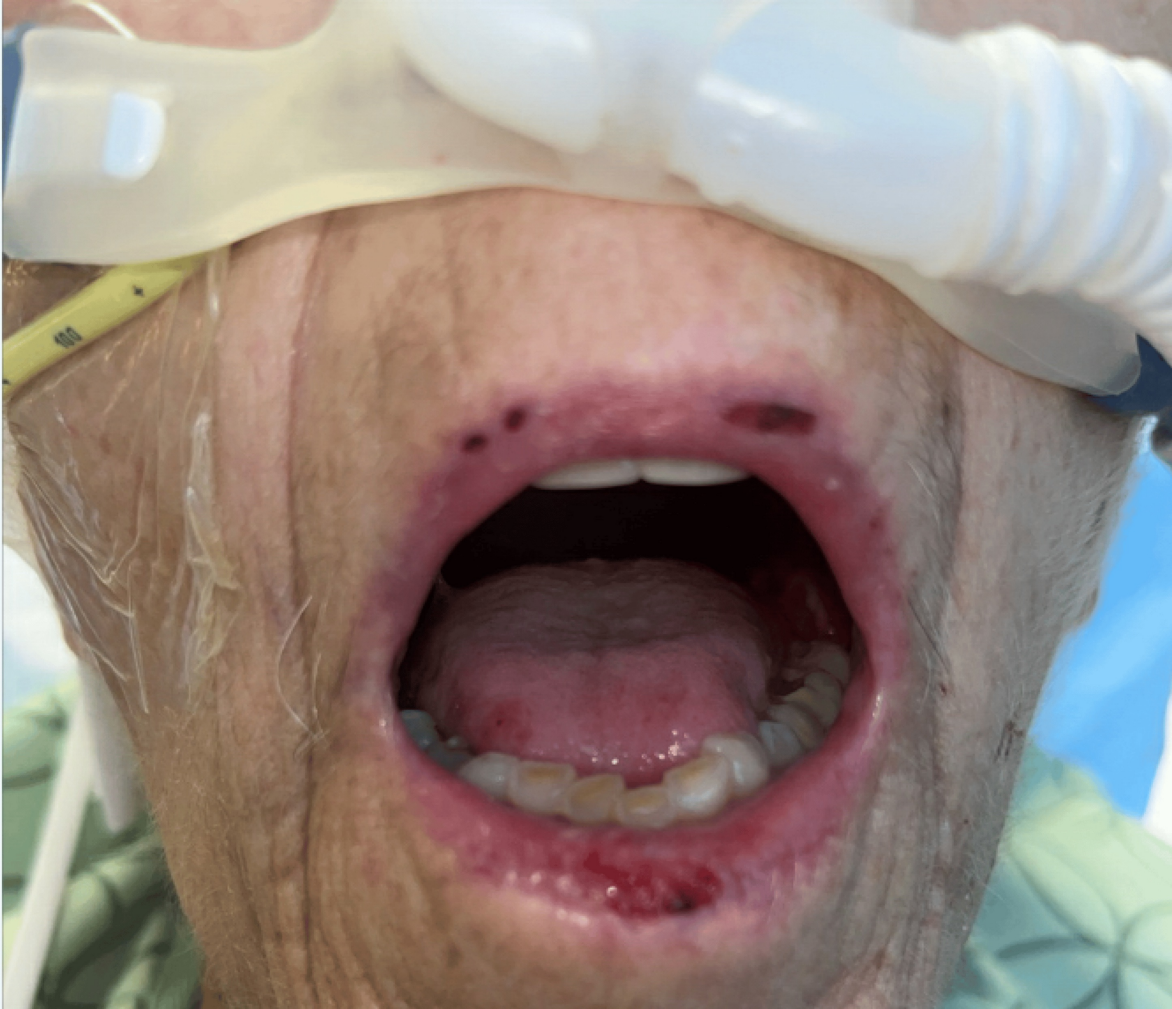
Oral mucositis in mucous membrane pemphigoid Severe ulcerative mucositis involving the labial and buccal mucosa, characterized by erythema, friability, and shallow erosions consistent with active mucous membrane pemphigoid.

**Figure 2:**
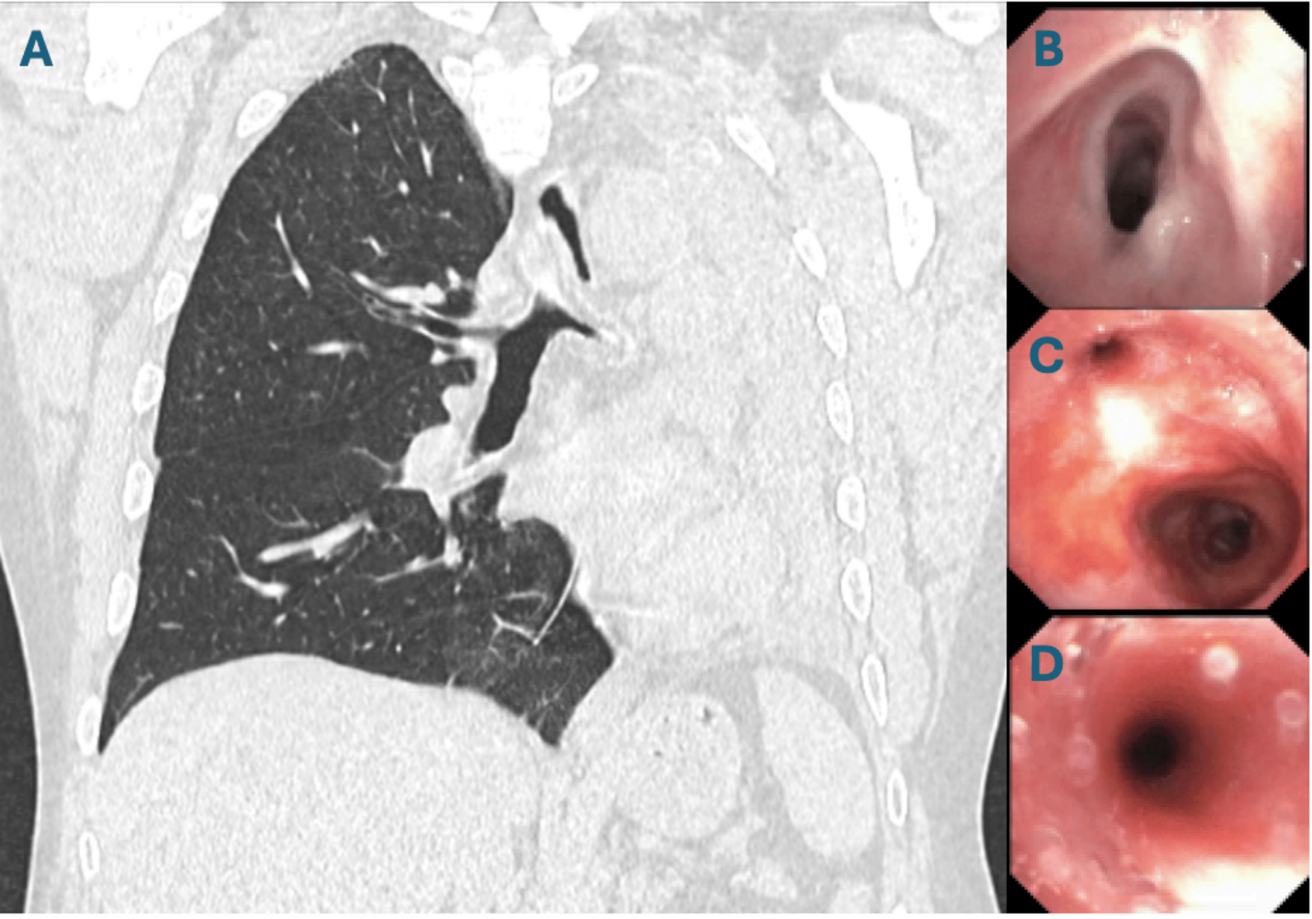
Imaging and bronchoscopic evidence of airway stenosis in mucous membrane pemphigoid (A) Coronal chest CT demonstrating complete collapse of the left lung with ipsilateral volume loss and mediastinal shift, caused by severe narrowing of the left main bronchus. (B) Bronchoscopic view of high-grade tracheal stenosis with circumferential mucosal thickening. (C-D) Progressive narrowing of the left main bronchus with friable, inflamed mucosa and near-complete luminal obstruction, consistent with advanced airway-predominant MMP.

**Figure 3:**
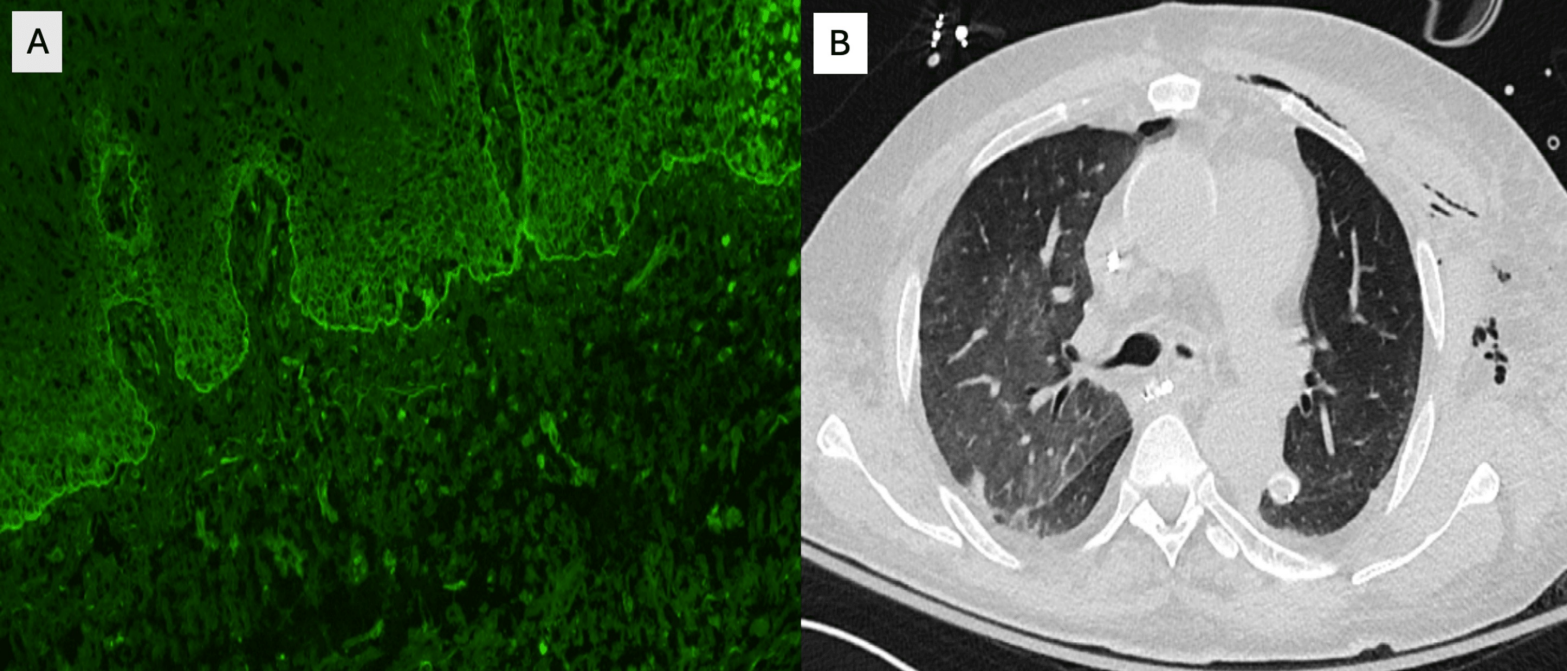
Post-intervention improvement and immunopathologic confirmation of mucous membrane pemphigoid (MMP) (A) Chest radiograph demonstrating improved aeration of the left lung following bronchoscopic intervention, with re-expansion of previously collapsed lung parenchyma. (B) Direct immunofluorescence (×200) of perilesional oral mucosa showing linear deposition of C3 and IgA, with lesser IgG, along the basement membrane zone—findings diagnostic of IgA-predominant mucous membrane pemphigoid.

A 62-year-old man with remote biopsy-confirmed MMP presented with progressive respiratory and mucocutaneous symptoms. In his initial disease course years earlier, he developed severe oral ulcerations and laryngeal involvement that progressed to upper-airway stenosis requiring tracheostomy. After receiving corticosteroids and rituximab, he entered remission, his tracheostomy was decannulated, and he remained stable for years without immunosuppression. Over subsequent years, he developed recurrent painful oral mucositis (Figure 1), hoarseness, ineffective cough, mucus retention, and intermittent dyspnea. Several corticosteroid tapers produced only transient improvement. His symptoms gradually worsened with the onset of dysphagia, hemoptysis, weight loss, and escalating dyspnea suggestive of recurrent cicatricial airway disease. During an acute decompensation marked by severe dyspnea and hypoxemia, chest imaging revealed complete opacification of one hemithorax with mediastinal shift (Figure 2A), consistent with collapse secondary to high-grade central airway obstruction. Urgent bronchoscopy demonstrated severe cicatricial subglottic stenosis and diffuse tracheobronchial mucosal inflammation. The mucosa appeared friable and easily sloughed, and the left main bronchus was nearly occluded by circumferential translucent fibrotic membranous tissue (Figures 2B-2D). These findings were characteristic of advanced airway-predominant MMP. Therapeutic bronchoscopy, including balloon dilation, cryotherapy, and mechanical removal of obstructing tissue, temporarily restored airway patency and allowed partial re-expansion of the collapsed lung (Figure 3A). However, each intervention yielded only brief improvement, with rapid restenosis despite systemic therapy, reflecting the aggressive cicatricial remodeling inherent to airway MMP. Given the rapid progression, a multidisciplinary team initiated intensive immunomodulatory therapy. The patient received pulse-dose intravenous methylprednisolone followed by high-dose oral corticosteroids. No alternative infectious, autoimmune, or structural cause explained the destructive airway scarring. Biopsies from oral and airway mucosa demonstrated subepithelial blistering with eosinophilic spongiosis. Direct immunofluorescence (DIF) revealed linear C3 and IgA deposition with weaker IgG along the BMZ (Figure 3B), consistent with IgA-predominant MMP. Indirect immunofluorescence showed low-titer circulating IgG. Antinuclear antibody (ANA) and ribonucleoprotein (U1-RNP) were weakly positive but clinically nonspecific. Despite aggressive immunosuppression and repeated bronchoscopic interventions, the patient developed refractory hypoxemic respiratory failure due to relentless central airway obstruction and ultimately died after a prolonged intensive care course.

## Discussion

Airway involvement in MMP is uncommon but associated with disproportionately high morbidity and mortality [6]. Although fewer than 10% of patients develop laryngeal or tracheal lesions, airway involvement can cause progressive cicatricial narrowing, leading to life-threatening obstruction [3,5,7]. Expression of BMZ antigens, such as BP180 and laminin-332, in bronchial epithelium provides a biological rationale for lower-airway involvement [2]. The POPS review described 11 cases of biopsy-confirmed tracheobronchial MMP, highlighting nearly universal mucosal inflammation, friability, circumferential scarring, and high-grade airway stenosis with mortality approaching 45% [6]. Our patient exhibited the hallmark features of airway-predominant MMP: recurrent mucosal flares, friable tracheobronchial mucosa, circumferential fibrotic occlusion, and rapid restenosis after intervention. Similar to the case described by Jalil et al., repeated bronchoscopic debulking and dilation produced transient improvement but failed to prevent rapid restenosis, reflecting the aggressive cicatricial biology of airway MMP [8]. Diagnosis can be challenging. Airway biopsies frequently sample inflamed or scarred mucosa lacking intact BMZ, resulting in false-negative DIF. Consistent with prior reports, our diagnosis relied on perilesional oral mucosal biopsy demonstrating linear C3 and IgA deposition [6,9]. Serologies are often negative or low-titer, further contributing to diagnostic delay [3].

Management requires combined systemic immunosuppression and interventional bronchoscopy. Corticosteroids remain first-line; steroid-sparing agents, such as mycophenolate, azathioprine, cyclophosphamide, or rituximab, are recommended for refractory disease [6,10]. Once fixed fibrosis develops, however, medical therapy alone is insufficient, and interventional bronchoscopy is central to maintaining airway patency. The American College of Chest Physicians endorses therapeutic bronchoscopy, including dilation, cryotherapy, mechanical debulking, and stenting for benign central airway obstruction [11]. Balloon dilation reliably restores luminal diameter with a favorable safety profile, although restenosis commonly necessitates repeat procedures [12,13]. Its effectiveness declines after multiple sessions, prompting the need for adjunct modalities [14]. Cryotherapy serves as an important complement. By preserving cartilage and promoting less fibrotic healing, both contact cryotherapy and spray cryotherapy have demonstrated safety and benefit in benign tracheal stenosis [15,16]. Spray cryotherapy has shown particular efficacy in circumferential scar tissue and often prolongs the intervention-free interval when combined with balloon dilation [17-19]. Mechanical debulking includes cold instruments, electrocautery, argon plasma coagulation, or laser, which remains essential for dense or obstructive scar webs and is frequently paired with dilation and cryotherapy [12]. Despite these options, restenosis is common in immune-mediated cicatricial disorders such as MMP [20]. Our patient’s rapid recurrence after each intervention mirrors patterns described in benign stenosis literature and reflects advanced fibrotic remodeling [6,8].

Delayed recognition is a major driver of poor outcomes. The POPS review reported a median delay of nearly two years between onset of respiratory symptoms and definitive bronchoscopy, with survival after diagnosis often limited to several months [6]. Our patient similarly presented late, when stenosis was already rapid and irreversible.

## Conclusions

Airway-predominant MMP is a rare but severe manifestation characterized by progressive mucosal inflammation, scarring, and recurrent central airway obstruction. Diagnostic delays are common due to the low sensitivity of airway biopsies and nonspecific serologies. Although bronchoscopy can provide temporary relief, rapid restenosis often occurs once fibrosis is established. Early recognition of airway symptoms and prompt multidisciplinary evaluation, including dermatology, pulmonology, and otolaryngology, are essential to prevent irreversible stenosis and reduce mortality.
